# Beyond the dental clinic: ending the global stagnation in pediatric caries with program science

**DOI:** 10.3389/fpubh.2026.1897048

**Published:** 2026-08-18

**Authors:** Smita Kumar, Saurabh Verma, Richa Khanna, Vikram Khanna, Hardeep Singh Malhotra, Raghuwar Dayal Singh, Sumit Kumar

**Affiliations:** 1 Program Science- Based Research, University of Manitoba, Winnipeg, MB, Canada; 2 Indian Health Action Trust, Lucknow, India; 3 King George's Medical University, Lucknow, India; 4 Humanex Technologies Solutions, Abu Dhabi, United Arab Emirates; 5 Department of Paediatric and Preventive Dentistry, King George's Medical University, Lucknow, India; 6 Department of Oral Medicine and Radiology, Faculty of Dental Sciences, King George's Medical University, Lucknow, India; 7 Department of Neurology, King George's Medical University, Lucknow, India; 8 Multi-Disciplinary Research Unit-Department of Health Research, King George's Medical University, Lucknow, India

**Keywords:** commercial determinants of health, early childhood caries, health equity, implementation science, medical-dental integration (MDI), program science, proportionate universalism, public health policy

## Abstract

Globally, early childhood caries is the most prevalent disease in children, even after a century of development. This demonstrates an “effectiveness illusion” where clinical methods fail to deliver true equity. The reason for this inconsistency arises from “dental exceptionalism”, which excludes oral health from the systemic medical care systems. Through this article, we advocate moving from a reactive, tools-focused Clinical Science model to a proactive, population-specific Program Science approach. By utilizing the principle of proportionate universalism, prevention can be integrated into the children's natural environment. We present an operational framework to guide Medical-Dental Integration, emphasizing the upskilling of primary care workforces and the use of real-time programmatic data to iteratively resolve implementation bottlenecks. Furthermore, we address structural barriers, including the commercial determinants of health, macro-economic sugar policies, and the health system feasibility, data governance, and financing shifts required to transition from fee-for-service models to value-based care. Ultimately, this framework provides actionable pathways for low-, middle-, and high-income health systems to achieve equitable, sustainable pediatric oral health outcomes.

## Introduction

For nearly a century, the worldwide dental community has maintained a long-standing belief that eradicating Early Childhood Caries (ECC) is essentially a matter of improved clinical detection. After investing in substantial funding and years of persistent research in perfecting the toolset, such as evidence of the efficacy of fluoride varnish, glass ionomer sealants, and silver diamine fluoride (SDF) (1), ECC persists as the most common non-communicable disease among children, globally affecting more than 530 million children.

This disparity reveals the effectiveness illusion. The concept of the “effectiveness illusion” corresponds to what implementation science formally characterizes as the “efficacy-to-effectiveness gap” (2) or systemic “voltage drop” (3). In this perspective, we apply the conceptual framework of effective illusion to describe the widening discrepancy between an intervention's high efficacy under tightly controlled settings and its diminished impact when deployed in real-world populations. Controlled clinical trials show that interventions such as dental sealants or SDF effectively prevent decay under strict monitoring. However, deploying these tools in complex health systems without addressing social inequities causes a significant voltage drop. This gap persists because traditional clinical models assume linear delivery pathways and underestimate social gradients, transport barriers, and commercial food environments that shape real-world accessibility and adherence.

This systemic implementation gap is heavily driven by the phenomenon we characterize here as Dental Exceptionalism (4), which is the separation of dental care from broader systemic, social, and economic health factors. This professional barrier separate oral health from broader public health, limiting effectiveness when relying solely on clinical settings.

ECC patterns show social gradients: high-income countries have stable caries rates, while indigenous, migrant, and low-socioeconomic groups face higher disease prevalence due to systemic barriers and low health literacy. Low- and Middle-Income Countries (LMICs) face challenges such as workforce shortages and inadequate rural dental infrastructure (5).

In resource-limited settings, a Program Science (PS) approach that emphasizes low-tech, high-impact methods, such as SDF and Atraumatic Restorative Treatment (ART), is practical. It should be assessed alongside factors such as the lack of clean water, changing eating habits toward refined, sweet foods, and cultural beliefs about baby teeth or milk teeth. Effective integrated models include school-based toothbrushing initiatives with fluoridated toothpaste in Southeast Asia (6) and joint child vaccination days in Latin America (7). These examples show that integrating oral health into existing public health efforts can improve results without requiring additional clinical facilities.

Similarly, nationwide taxes on sugary drinks in countries like Mexico (8) and the UK (9) show that commercial influences can be adjusted to act as public safeguards, supporting local actions like dental sealants.

Health systems lack a strategy to coordinate structural factors influencing caries development. Overcoming this inertia requires shifting from rigid methods to the multi-sectoral Program Science (PS) framework (10).

## The core principles of program science

Program Science (PS) is an iterative, mathematical, and operational approach that optimizes the selection, sequencing, and delivery of public health interventions by directly embedding scientific inquiry within the program lifecycle. Originally conceptualized by Blanchard and Aral (11) to address the complex, population-level dynamics of HIV and sexually transmitted infections (STIs), the transferability of PS to pediatric oral health is rooted in its ability to address diseases with steep social gradients and complex behavioral-environmental etiologies. To understand its utility, PS must be explicitly distinguished from related health-system methodologies, including Implementation Science (12), Quality Improvement (QI) (13), and Learning Health Systems (LHS) (14).

Implementation science primarily operates from a top-down approach, focusing on the “how-to” of integrating a specific, pre-determined clinical tool into a specific setting. PS, conversely, begins with population-level epidemiology, asking which combination of interventions is optimal for this specific population at this specific time, actively evaluating the collective impact of an entire program portfolio (15). QI methodologies focus on micro efficiencies, such as reducing clinic wait times, whereas PS functions at a macro level, regulating policies and resources to promote equity and public health. LHS offers data infrastructure, with PS translating insights into real-time adjustments. Beyond HIV/STI, PS has been adapted for maternal and child health (16) in LMICs, improving pathways and lowering neonatal mortality through data-driven approaches.

While frameworks such as the Consolidated Framework for Implementation Research (CFIR) and the RE-AIM (Reach, Effectiveness, Adoption, Implementation, Maintenance) model are useful for analyzing organizational factors in the adoption of clinical interventions, they often operate within bounded structures (17). In contrast, PS offers a macro-systemic advantage for pediatric oral health by treating multiple interventions as a dynamic, continuous system driven by population-level epidemiology. Instead of focusing narrowly on optimizing a single tool (such as fluoride varnish application) within an existing clinic, PS provides the operational machinery to strategically sequence, allocate, and scale multi-level clinical, behavioral, and structural interventions in direct alignment with the shifting social risk profiles of an entire community.

## The program science operational framework for pediatric oral health

The PS concept can be translated into a structured, point-wise, achievable milestone for pediatric oral health. The schema is executed in four stages to control and eliminate ECC, from the appointment-based individual visit to the pediatric dental surgeon/dental office to community-focused care, using population-based data to plan, healthcare guidelines to act, and defined field feedback to improve services.

### Phase 1: Strategic assessment (epidemiology and social risk mapping)

The first phase in the schema of PS is to evaluate the pediatric population with clinical assessment, which includes details of the patient's oral health status, Decayed Missed Filling (dmft) indices, and gingival and periodontal health indices for proper risk mapping. Utilizing the public health systems to incorporate geographic registries, housing stability, food security, socioeconomic profiling, along with oral health information. Analyzing the social determinants helps to identify the community at risk and vulnerable children at high risk of dental caries. This mapping helps guide public health efforts to address the social and environmental factors that affect ECC in specific areas and populations/communities, avoiding the random distribution of resources.

### Phase 2: Program design (proportionate universalism)

The intervention strategies are designed and developed based on the epidemiological data collected in Phase 1, guided by the principle of Proportionate Universalism (18). This approach addresses the shortcomings of implementing uniform strategies and the stigma that can accompany targeted interventions by implementing a two-tiered system. This design ensures that every child receives fundamental preventive oral health education and screening. Advanced methods, including mobile SDF application, subsidized fluoride treatments, and nutritional programs, are assigned based on social risk factors and disease severity identified during evaluation, ensuring resources meet community needs.

### Phase 3: Implementation and integration (medical-dental integration)

In this phase of the PS schema, the customized intervention approaches are implemented within the existing pediatric healthcare system. This strategy aims to break down the professional barrier of dental exceptionalism (4). The phase primarily aims to expand preventive pediatric care by educating primary care pediatricians, family physicians, and community nurses. The general medical exam, school health checkup, and early childhood immunization should include a basic oral health examination, visual caries screening, and fluoride varnish application.

The intervention strategies are designed to effectively deal with the barriers related to location, cultural differences, financial issues, and also the constraints that often hinder special dental appointments with general or pediatric dentists. By utilizing the established healthcare facilities, the child is scheduled to visit as part of the routine healthcare system, and the primary healthcare provider can serve as the first line of defense against ECC.

### Phase 4: Real-time optimization (rapid-iteration feedback loops)

To ensure the success of the framework in long-term evaluation, it is necessary that the proposed methodology be evaluated in parallel with the real-world execution of the intervention. Rapid, programmed feedback iteration cycles are devised and managed for this. Realtime data capture and simultaneous operational evaluation with control points are fixed at the field level. This leads to the evaluation of the framework rather than the conventional method of retrospective re-evaluation of protocols, which usually emerges later. Red flags raised through real-time data checks help to rectify many issues, such as participant engagement hindered by complicated administrative parental consent processes, school scheduling conflicts, or transportation limitations, leading to quick and adaptive modifications.

The framework is made robust with this operational approach, transforming the schema of PS into an adaptive, self-regulating public health system that improves delivery effectively, addressing the systematic hindrances and obstacles, and ensuring equitable oral health outcomes across the population.

## Breaking the barriers of inequality in geography

The dental clinic presents structural accessibility barriers, resulting in highly disparate treatment across underserved populations. For families living in poverty, the barriers to reaching that chair, including transportation costs, time off work, low health literacy, and the psychological weight of the “medical model”, can be impossible to overcome. Program Science advocates for “de-territorializing”, i.e., the strategic relocation of preventative healthcare interventions away from traditional, fixed clinical spaces into accessible community-based touchpoints, such as geographically appropriate health care delivery units, schools, and community centers.

This requires a shift from “Universalism” to “Proportionate Universalism” (18). Traditional public health often provides everyone with the same level of care, but in an unequal society, this can widen the gap, as those with greater social capital can access services more easily. Proportionate Universalism maintains a baseline for everyone but intensifies care for those at the highest social and biological risk; it, thus, flattens the social gradient and aims at equity of outcomes.

This can be accomplished by prioritizing the PS approach in resource-limited settings, including LMICs in the Global South, as well as disadvantaged populations in high-income countries. Merging dental records with housing stability and food insecurity data could help identify children who are waiting to be seen in emergency departments for complications. Alternatively, mobile units and community health workers can be assigned directly in high-prevalence areas.

## The mandate for integrating medical and dental care

Discriminating mouth from the body is a historical irregularity, not a clinical need. Nowadays, there exists a fragmented care delivery dichotomy, where a pediatrician will examine a child's heart, lungs, and ears, but ignore decaying teeth right next to them. Therefore, there is an imperative need for Medical-Dental Integration (MDI), which mandates the integration of oral health into the routine medical care (19).

As pediatricians are the first point of care for children under five, upskilling their staff to apply fluoride varnish and conduct evaluations can reach far more children than a conventional dental office ever could. Moreover, the “de-professionalization” of prevention must be accepted. There should not be a requirement for a surgeon to apply varnish or speak to a parent about sugar. By delegating to community health workers, dental therapists, hygienists, and nurses, care could be made economical, reasonable, and culturally equitable. The purpose is that the dentist's chair must be used for routine monitoring and health maintenance, not for chronic care management.

## Real-time learning

A conventional PS point of view discards the slow, retrospective assessment usual in public health and instead seeks to address bottlenecks and logistical issues promptly. Generally, a program is evaluated years after it starts, too late for the children who participated. Ideally, we need a continuously learning health system (20) built on realistic iteration. For instance, in a school, if a sealant program has a skipping rate below 50%, it's prejudiced to criticize parents as “defiant”. A Program Scientist looks into the reasons for the shortfall in the effectiveness of communication and guidance, a confusing consent form, or any other conflict. By rapidly addressing these logistical obstacles, the program can be tweaked to the community's needs rather than following a rigid, potentially ineffective policy.

## Addressing the commercial foundations of disease

Furthermore, we analyze the structural propagation of ECC through what can be termed the “commercial foundation of disease”, examining how market drivers and political economies of ultra-processed, high-sugar foods fundamentally shape population risk profiles prior to individual clinical exposure. We usually pay no attention to this, instead blaming individual behavior. It is not justified to expect a parent to curb high-sugar, market-driven, ultra-processed foods.

We encourage dental professionals to rise above the operatory and take part in policy-making. We need to support macroeconomic management tools, such as taxes on sugar-sweetened beverages. We can follow the models from Mexico and the UK, which exemplify the effectiveness of these taxes in reducing consumption and eventually persuading manufacturers to modify their products (8, 9). According to PS, a sugar tax serves as a safeguard compared to dental sealants.

## Scalability challenges in the global south

In LMICs, dental infrastructure is usually non-functional, especially in rural regions. There, PS approach represents a critical operational imperative. Within these environments, we must emphasize low-tech, profound-effect interventions such as SDF and Atraumatic Restorative Treatment (ART), as these treatments don't require electricity, running water, or expensive drills. These are the practical solutions provided by trained community workers in a schoolroom or a village hut. By aiming to prevent decay rather than drilling and filling, we can provide equitable care.

## The economic shift: paying for health, not drills

Amongst others, an impediment to this vision is the “Fee-for-Service” (FFS) payment system, which rewards quantity over quality; it pays for the course of action rather than preventing the cavity. This is economically impaired (21). One sitting of general anesthesia for a child with severe ECC is pricier than rendering preventive varnish to an entire school annually.

We should switch to value-based care programs that help establish a sustainable healthcare system. When monetary rewards are linked to the child's health, hurdles to community outreach and medical integration wane.

## Health-system readiness, implementation barrier, heterogeneity, and phased operational pathways

Integrating preventive oral healthcare into routine primary pediatric care can be an icebreaker. To make this more feasible, short, consistent upskilling programs should be introduced. The roles of dental auxiliaries and community health care workers are of utmost importance and may involve managing and overcoming regulatory hurdles often associated with dental associations. This requires approval from the regulatory bodies for the data management. Successfully scaling this program requires structural revisions to professional scope-of-practice regulations for allied health professionals, updating institutional accreditation standards for primary care clinics to incorporate oral health infrastructure, and modifying the national public health policies that actively legalize and incentivize clinical task-shifting.

Implementing the PS framework varies across countries due to differences in health system structure, finances, and workforce capacity. A localized, phased approach is necessary, tailored to each setting. In LMICs facing workforce shortages and limited rural infrastructure, immediate deployment of MDI via electronic registries is impractical. Instead, a phased plan is recommended: Phase 1 emphasizes low-cost community activities like training health workers in SDF application and school-based fluoridation. After establishing these, Phase 2 integrates visual oral triage into primary care. In high-income countries with advanced digital systems but marginalized groups, the main obstacle is institutional fragmentation. Here, Phase 1 should prioritize data governance by linking dental and medical Electronic Health Records with social risk data, followed by Phase 2′s shift from fee-for-service to value-based financing. Understanding these differences ensures the PS framework remains flexible and scalable.

## Conclusion

**Figure 1:**
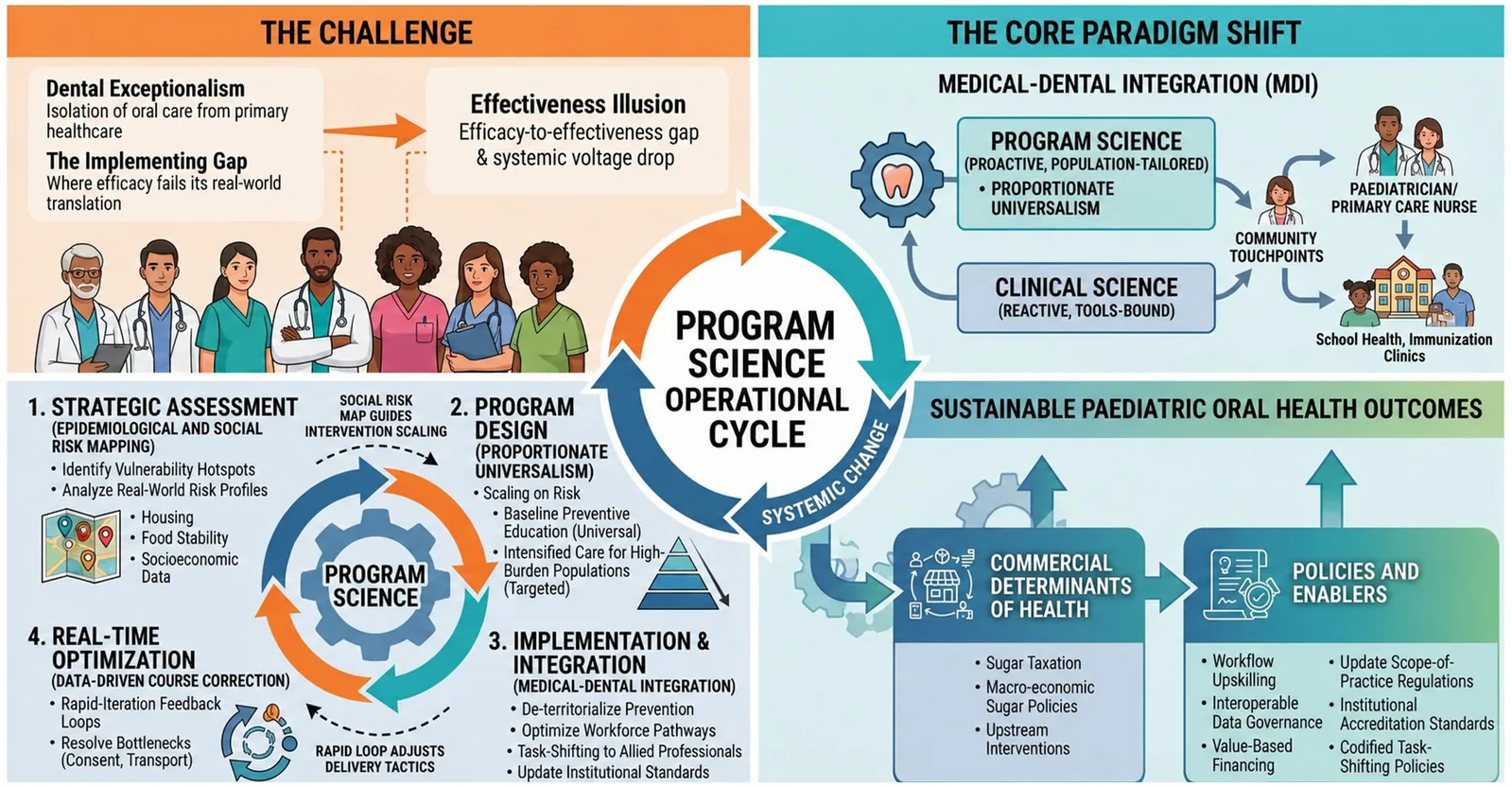
The program science operational framework and systemic paradigm shift for paediatric oral health. This conceptual infographic outlines the structural transformation required to shift from traditional, clinic-bound dental models to an integrated public health strategy to address the global stagnation of early childhood caries (ECC). The framework illustrates the continuous, self-correcting four-phase loop of the program science operational cycle. Phase 1: Strategic assessment: utilizes epidemiological and social risk data to map community vulnerability hotspots. Phase 2: Program design (proportionate universalism): establishes the direct operational link between mapped social determinants and intervention intensity. Phase 3: Implementation and integration: focuses on de-territorializing prevention through Medical-Dental Integration. Phase 4: Real-time optimization: employs rapid-iteration data feedback loops to bypass field bottlenecks and administrative constraints. Macroscopic policy drivers, including workforce upskilling, modernized scope-of-practice regulations, value-based capitated financing, and upstream commercial sugar interventions, are depicted as structural enablers sustaining this equity-driven paradigm.

The persistent global burden of ECC reveals an implementation gap primarily driven by institutional and policy issues, rather than a lack of clinical expertise. While highly effective diagnostic and preventive tools are available, their public health impact remains constrained by the historical segregation of oral healthcare from primary medical systems. The evidence reviewed in this article indicates that a PS framework offers a plausible, systematic pathway to address this stagnation. Rather than attributing intervention failures to individual or family non-compliance, this framework proposes that health systems should be held accountable for operational accessibility and equity of delivery. To align effectively with the World Health Organization's 2030 global oral health goals, health systems should apply the same level of operational rigor to delivery pathways that has historically been dedicated to clinical materials. Consequently, we propose a strategic reallocation of preventive interventions from isolated clinical spaces to community-anchored care touchpoints, integrating oral care into the natural environments where children live, learn, and develop (Figure 1).

## Data Availability

The original contributions presented in the study are included in the article/supplementary material, further inquiries can be directed to the corresponding author.
